# Comparing independent prescribing to patient group direction use in a general practitioner out-of-hours service: a retrospective cross-sectional service evaluation

**DOI:** 10.29045/14784726.2024.9.9.2.21

**Published:** 2024-09-01

**Authors:** Hayley Stevens, Beryl Mansel, Jayne Cutter

**Affiliations:** Welsh Ambulance Services NHS Trust ORCID iD: https://orcid.org/0000-0001-8624-5428; Swansea University ORCID iD: https://orcid.org/0000-0002-7747-3004; Swansea University ORCID iD: https://orcid.org/0000-0002-0494-8777

**Keywords:** advanced practice, advanced practitioner, independent prescribing, non-medical prescribing, nurse, out-of-hours service, paramedic, patient group direction, verbal order

## Abstract

**Introduction:**

Global demand for healthcare is escalating, prompting exploration of innovative strategies to augment service capacity. Independent prescribing (IP) helps to address this challenge, allowing non-medical professionals to prescribe medication. Paramedics in the UK were granted prescribing privileges in 2018, yet uptake remains low. Despite qualitative evidence indicating that paramedic prescribing is beneficial, quantitative comparisons of medication provision between prescribers and non-prescribers are lacking. Paramedics provide patients with non-emergency medication by three different routes: IP, using a patient group direction (PGD) or with prescriber support.

Advanced paramedic practitioners who are not qualified as independent prescribers, rotating through ambulance and general practitioner out-of-hours services, offered an opportunity to quantitatively compare medication supply.

**Methods:**

This study compares medication supply by three advanced paramedic practitioners using PGDs with three prescribing nurses in a Welsh general practitioner out-of-hours service. A cross-sectional design was employed to retrospectively review electronic patient clinical records between 1 December 2019 and 30 November 2020, including patients presenting with one of five generalised clinical conditions (urinary, soft tissue, respiratory, abdominal pain, ear). Descriptive analysis and non-parametric tests compared medications prescribed or supplied, how patients received medication and reasons for seeking prescriber support.

**Results:**

A total of 397 patient records were analysed. Paramedics supplied medications more frequently with prescriber support (68.2%) than via PGD (27.9%). Nurses predominantly prescribed medication independently (99.3%). Medication provision was comparable when paramedics had prescriber support. Reasons for paramedic support-seeking included having no PGD available (34.1%) and PGD being excluded from use (28.4%).

**Conclusions:**

Advanced paramedic practitioner medication supply using PGDs and prescriber support was comparable to that of prescribing nurse colleagues. However, autonomy restrictions highlight the need for paramedic prescribing in services where prescriber availability is limited. Further research evaluating the efficiency and cost-effectiveness of PGD use versus IP is necessary. Additionally, the qualitative benefits of IP, such as improved patient care and satisfaction, warrant due consideration when implementing future healthcare strategies.

## Introduction

Global demand for healthcare is increasing; many countries cannot increase capacity to match. The World Health Organization’s (2016) need versus shortage forecasts indicate that by 2030, 2.3 million doctors will be required worldwide. Many countries have implemented independent prescribing (IP) to increase capacity and medication access (Abuzour et al., 2018).

In the UK several non-medical professionals now prescribe, with paramedics offered the opportunity in 2018 (Edwards et al., 2020). However, by 2021 only 3.2% of paramedics (1016) in the UK were registered as independent prescribers (Health & Care Professions Council, 2021). Thus far, no quantitative studies compare medication provision between paramedic prescribers and non-prescribers. A mixed-methods survey by Eaton et al. (2022) suggests that prescribing paramedics make diagnoses more often, and deal with more complex patients than non-prescribing paramedics. This indicates that prescribing increases clinician autonomy and service capacity (by increasing patient turnover) (Health Education Northwest, 2015), which NHS England (2015) anticipated as a main driver for implementation.

Evidence from other non-medical professions shows independent prescribers have higher patient satisfaction scores (Courtenay et al., 2015), provide more information (Carey et al., 2017; Van Der Biezen et al., 2016), spend more time deprescribing (Collins, 2018; Nuttall, 2018), reviewing medications and assessing adherence (Carey et al., 2017; Weeks et al., 2016) and have longer consultations (Carey et al., 2017; Courtenay et al., 2015; Smits et al., 2019; Van Der Biezen et al., 2016) than GPs or non-prescribing colleagues. The extra time taken to provide such prudent, preventative and high-quality healthcare could pay dividends in the long term by improving patients’ ill health and reducing reattendance.

Although research from other non-medical prescribers is reassuring, the lack of profession-specific research presents a barrier, as there is no evidence of where best to implement paramedic prescribing for maximum service benefit (Edwards et al., 2020). The College of Paramedics’ (2018) prescribing guidance suggests implementation in advanced practice roles that are able to evidence regular use of prescribing, which is of benefit to the patient. Paramedics in such roles should work at an advanced level, within strict governance structures, and should have access to regular medical mentorship (College of Paramedics, 2018). However, roles that meet this guidance are uncommon and yet to be evaluated.

Another potential barrier to implementation is cost. Non-medical prescribing courses cost between £1300 and £2700 (Health Education England, 2021; Swansea University, 2022), with prescribers attracting a band 7 (£40,057–£45,839) to band 8a wage (£47,128–£53,219) without enhancements (Courtenay et al., 2015, 2018; NHS Staff Council, 2022). This expenditure is especially significant when other mechanisms, such as patient group directions (PGDs), can be used to supply medication.

A PGD is a list of set and pre-defined criteria allowing the supply of a particular dose, duration and frequency of medication to a set patient group (National Institute for Health and Care Excellence (NICE), 2017a). When criteria are not met, the PGD cannot be used. In such circumstances a prescriber can be contacted for advice. An independent prescriber may also seek doctor advice when a patient’s presentation is out of their scope of practice or competence.

Advice-seeking in emergencies can result in a verbal order to supply or administer medication, where not doing so would compromise patient care (Royal Pharmaceutical Society, 2019). Verbal orders are more common when the patient is in a different location, and the logistics of providing a prescription would cause delays (Evans & Mullen, 2009). In such instances, the person issuing the verbal order takes over legal responsibility for the patient and the medication prescribed (General Medical Council, 2022). Advice-seeking may also result in a prescription being collected or faxed to a pharmacy. Overall, there are three different routes in which paramedics can provide patients with medication: IP by the clinician, following a PGD or with support from a prescriber.

Two comparative non-paramedic studies investigating PGD use reported how often advice was sought. Both Black et al. (2020) and Courtenay et al. (2015) found prescribing nurses worked more autonomously and required less support from GP colleagues than non-prescribing nurses or nurses using PGDs. Courtenay et al. (2015) conclude that the time to learn and then impart medication advice increased service costs. Comparing how often advice was sought and the reasons for this provides valuable information on the autonomy of PGD use and IP practice.

In the current study, paramedics with a Master’s degree in Advanced Clinical Practice (advanced paramedic practitioners) rotated between ambulance and general practitioner out-of-hours (GPOOH) service. Advanced paramedic practitioners and prescribing nurses will henceforth be referred to as ‘paramedics’ and ‘nurses’ for ease of reading.

The paramedics in this study supplied medication via PGD. Nurses in the same service prescribed medication. Both types of clinicians sought advice from a medical prescriber when needed. This working model created a novel opportunity to compare IP to current PGD practice in a setting that meets prescribing guidance set out by the College of Paramedics (2018).

## Methods

This study compares medication supply by paramedics using 19 PGDs with prescribing nurses in a GPOOH service. Our objectives were to:

- Compare medications prescribed or supplied by nurses and paramedics.
- Compare how patients receive medication (PGD, IP or with prescriber support).
- Compare the reasons for gaining prescriber support.

The study used a cross-sectional design to review electronic patient clinical records retrospectively. A purposive sample of three full-time nurse prescribers was selected. Three non-prescribing paramedics were randomly selected from a pool of six. Records were reviewed between 1 December 2019 and 30 November 2020.

Clinical-coding review identified the most frequently occurring conditions. These were too specific to compare individually, so they were combined into five more generalised clinical presentations, outlined in Table 1. This approach limited data to a manageable level. The sample was further limited to face-to-face consultations to reflect the independent use of PGDs.

**Table 1.** Condition codes combined into the top five patient clinical presentations.

Condition code	Clinical presentations (grouped condition codes)
Urinary tract infection Cystitis Acute pyelonephritis	Urinary
Skin/subcutaneous infection Insect bite – leg + infection	Soft tissue
Lower respiratory tract infection Upper respiratory tract infection	Respiratory
Abdominal pain	Abdominal pain
Otitis media Otitis externa Earache symptoms Ear symptoms	Ear

The top five clinical presentations from six clinicians generated 527 electronic patient clinical records. Of these, 130 were excluded due to missing demographic or medication data (e.g. frequency/dosage), leaving a final sample of 397 records.

Retrospective study data were collected by one researcher (HS) from January to March 2021. Electronic patient clinical records were assessed by the researcher to establish if the medications provided by paramedics met PGD criteria. Where data collection was subjective, such as deciding whether a PGD could have been used or not, cases were discussed by the researcher and a service clinical lead, with consensus reached on each occasion.

On the system clinicians use to create patient clinical records, a prescribing function automatically adds medication details to the record when selected from a drop-down list. To complete the record either ‘prescription issued’ or ‘no prescription issued’ is selected. From the ‘prescription issued’ tab, a prescription can either be generated or recorded as handwritten. Where a medication is dispensed from stock, management advice is to select ‘record as handwritten’ and note ‘dispensed’ in the free text. Alternatively, all medication details including ‘dispensed’ can be recorded in free text.

Only one of the three paramedics recorded ‘dispensed’. This could be accurate; however, due to the frequency of recording from the one paramedic, it seems more likely that the other two paramedics were perhaps unaware of this record-keeping practice. In these instances, it is difficult to tell if advice-seeking resulted in a verbal order to dispense medication or if a handwritten prescription was issued. In view of this, advice-seeking will henceforth refer to both verbal orders to dispense medication and when a prescription has been handwritten.

Quantitative data analysis was performed using Statistical Package for the Social Sciences (SPSS version 26) software once exported from the Microsoft Access Database (IBM Corp., 2019).

Data were not normally distributed (Kolmogorov-Smirnov p <0.000 and Sharpiro-Wilk: p <0.001 tests). Categorical data meeting the assumptions of the relevant test (PGD versus non-PGD medications) were compared using the non-parametric chi-squared test. Statistical significance was set at p <0.05. As nurses saw more patients than paramedics, all other data (sex, age range, number of patients per clinician group, clinical presentation, how patients receive medication, reason for seeking support) were analysed descriptively, with median or percentages reported.

### Ethical considerations

As a retrospective study with remote data collection, patient treatment, staff assessment and patient management were unaffected. The University Research Ethics Committee (151220) permitted the study. As a service evaluation, Health Research Authority approval was not required. GPOOH Service Management provided access to anonymised electronic patient clinical records. All patient, staff and hospital identifiers were removed prior to receipt of the data. Only staff profession was known to the researcher.

## Results

### The sample

Of the 397 patients, 62.2% (n = 247) were female and 37.8% (n = 150) were male. The median age of patients was 3 years (nurse median = 3 years, paramedic median = 5 years). The largest age range was 18 years and under (27.0%, n = 107); the smallest was 89–98 years (4.3%, n = 17).

Nurses saw almost three-quarters of patients (74.8%, n = 297). Paramedics saw a quarter (25.2%, n = 100).

Urinary presentations were most common for paramedics (35.0%) and nurses (31.7%). Abdominal pain was least common (paramedics = 7.0%, nurses = 7.7%). Table 2 details clinical presentation and frequency.

**Table 2.** Number of patients per clinical presentation.

Clinical presentation category	Paramedic patients	Nurse patients	Total
Urinary	35 (35.0%)	94 (31.7%)	129 (32.5%)
Respiratory	33 (33.0%)	54 (18.2%)	87 (21.9%)
Soft tissue	15 (15.0%)	62 (20.9%)	77 (19.4%)
Ear	10 (10.0%)	64 (21.5%)	74 (18.6%)
Abdominal pain	7 (7.0%)	23 (7.7%)	30 (7.6%)
Total	100 (100%)	297 (100%)	397 (100%)

### Medications prescribed or supplied

A total of 584 medications were given to 397 patients. Paramedics gave 129 medicines. Nurses gave 455. Of the 584 medications, there were 66 different types: 17 were medications covered by a PGD, and 49 were non-PGD medications. Despite the smaller range of PGD medications, these 17 were prescribed or supplied most frequently by paramedics (76.7%) and nurses (69.5%). Table 3 details the frequency of PGD medications prescribed or supplied.

**Table 3.** Frequency of patient group direction medications prescribed or supplied by paramedics and nurses.

Patient group direction medication	Paramedic	Nurse	Overall total	Patient group direction medication	Paramedic	Nurse	Overall total
Amoxicillin	32 (7.7%)	78 (18.8%)	110 (26.5%)	Ibuprofen	2 (0.5%)	9 (2.2%)	11 (2.7%)
Co-amoxiclav	10 (2.4%)	42 (10.1%)	52 (12.5%)	Paracetamol	0 (0.0%)	6 (1.4%)	6 (1.4%)
Flucloxacillin	11 (2.7%)	40 (9.6%)	51 (12.3%)	Diazepam	4 (1.0%)	1 (0.2%)	5 (1.2%)
Nitrofurantoin	14 (3.4%)	27 (6.5%)	41 (9.9%)	Phenoxyme-thylpenicilln	0 (0.0%)	3 (0.7%)	3 (0.7%)
Prednisolone	6 (1.4%)	22 (5.3%)	28 (6.7%)	Dioralyte	0 (0.0%)	3 (0.7%)	3 (0.7%)
Trimethoprim	1 (0.2%)	24 (5.8%)	25 (6.0%)	Cetirizine	0 (0.0%)	1 (0.2%)	1 (0.2%)
Co-codamol	4 (1.0%)	19 4.6%)	23 (5.6%)	Loperamide	1 (0.2%)	0 (0.0%)	1 (0.2%)
Doxycycline	5 (1.2%)	16 (3.9%)	21 (5.1%)	Tetracaine	0 (0.0%)	0 (0.0%)	0 (0.0%)
Clarith-romycin	6 (1.4%)	12 (2.9%)	18 (4.3%)	Fluorescein	0 (0.0%)	0 (0.0%)	0 (0.0%)
Prochlor-perazine	3 (0.7%)	13 (3.1%)	16 (3.8%)	Total (%)	99 (23.8%)	316 (76.0%)	415 (99.8%)

Note: Percentages are correct to one decimal place and therefore do not add up to 100%.

**Figure 1:**
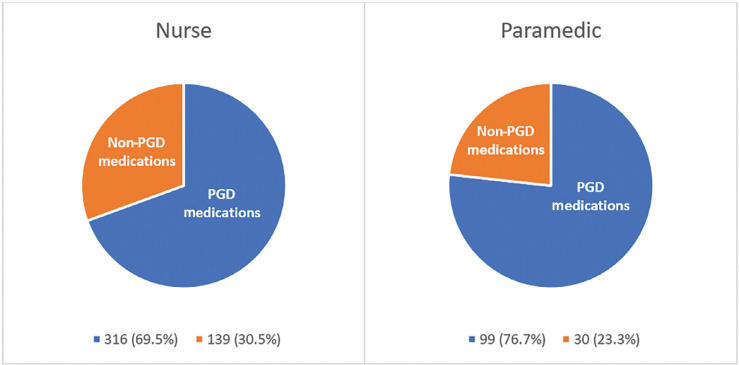
Figure 1. A group comparison of patient group direction and non-patient group direction medications prescribed or supplied.

Figure 1 compares the number of PGD medications to the number of non-PGD medications per group.

Although nurses prescribed non-PGD medications (n = 139) more frequently than paramedics supplied them with support (n = 30), the difference was not statistically significant (p = 0.133).

### How patients received medication

How patients received medication was compared: that is, PGD, IP or with prescriber support. Paramedic medications were supplied more often with prescriber support (68.2%, n = 88) than by PGD (27.9%, n = 36). On five occasions (3.9%), it was unclear if a PGD was utilised or if support was sought.

Nurses prescribed 99.3% of the time (n = 452) and gained three verbal orders (0.7%).

### Reasons for gaining prescriber support

Of the paramedic medications, 68.2% were supplied with prescriber support. Looking at this subset of data, the most common reason for a paramedic to seek prescriber support was that there was no PGD available for the medication supplied (34.1%, n = 30). A PGD was available 28.4% of the time (n = 25), but exclusion criteria prevented medication provision. Prescriber support was gained 25.0% of the time (n = 22) when a PGD could have been used independently. In 11 cases, there was insufficient information to decide if a PGD could be used independently or if prescriber support was indicated (12.5%). Two of the three verbal orders (0.7%) received by nurses resulted from referring children to the ear, nose and throat department, who advised antibiotics until the child could be seen routinely. The third verbal order resulted from seeking GP advice regarding hypertension medication. These were the only times that a referral resulted in a verbal order.

## Discussion

### The sample

The most common age (27.0% <18 years) and sex (62.2% female versus 37.8% male) distributions of the sample are slightly different from the resident population (19.8% <18 years and 50.9% female versus 49.1% male) (Welsh Government, 2020), probably as there is a lower threshold for seeing children face to face (Department of Health, 2003) and as women are more likely to seek medical attention than men (Wang et al., 2013).

An English GPOOH study by Collins (2018) also found urinary, respiratory, soft-tissue and abdominal pain to be the most common presentations. Additionally, the current study found ear complaints common, possibly as the sample was not restricted to home visits. Ear presentations are more frequent in children (Hughes, 2018), who are usually mobile, not requiring a home visit. The commonality of aliments and distribution of patient demographics in the sample appears true to a GPOOH workload.

As nurses prescribe remotely, they often remained in the assessment centre, conducting telephone consultations and assessing mobile patients. The mobility of patients with ear complaints is likely why the nurses saw more of these patients than the paramedics (10.0% versus 21.5%). Equally, as paramedics are accustomed to home visiting, they likely chose, or were allocated to, breathless patients unable to travel more often than the nurses (33.0% versus 18.2%).

Personal preference, qualifications, confidence and experience influenced the clinical presentations clinicians chose to see. The extent of this impact on the results is unknown and likely increased when comparing clinicians from different professions. Future studies should focus on within-profession comparison, where possible, to limit unmeasured variables influencing results.

### Medications prescribed or supplied

Paramedic leads selected the 19 PGD medications specifically for their usefulness and frequency of use. Therefore, it is unsurprising that 17 of these were most frequently prescribed or supplied by nurses and paramedics (76.7% versus 69.5%). The remaining two, tetracaine and fluorescein, were not indicated for use in the sample.

Interestingly, for non-PGD medications, no statistical difference was found between the number of medications prescribed by nurses and the number given by paramedics with support (30.5% versus 23.3%, p = 0.133). These results suggest that PGD use, alongside good prescriber support, allows paramedics to supply patients with a similar level of medication as their prescribing colleagues. This data may interest services implementing PGDs, where a good level of medical support is available.

However, the sheer volume (n = 169) and range of non-PGD medications (49 different types) indicate that without prescriber support, the current 19 PGDs are ineffective in meeting patient demand in this service – especially when, in practice, many more patient ailments than the limited sample would require medication provision.

In keeping with findings in a previous study (Black et al., 2020), the use of PGDs places high demand on prescribing colleagues. This is a fundamental consideration for services where verbal order or remote support is not practised, where there are prescriber shortages or where there is a need for a larger, more autonomous workforce.

### How patients received medication

Paramedics sought prescriber support more frequently than nurses (0.7% versus 68.2%), despite paramedics seeing only 25.2% of patients. A high level of prescriber support has cost and efficiency implications. Courtenay et al. (2015) found non-prescribing nurses waited for a prescription to be signed or discussed medication options with a GP significantly more often than prescribing nurses; such activities lengthen consultations. This slows access to medication (Bedson & Latter, 2018; Courtenay et al., 2015), reduces patient turnover and increases costs (Health Education Northwest, 2015).

Previous literature suggests that a five-minute advice-seeking episode costs approximately £19 in GP time alone (Courtenay et al., 2015; Curtis, 2013). While the length of the 88 advice-seeking episodes in this study was not measured, assuming a five-minute length would equate to more than seven hours of GP time at a cost of £1672, quadrupling to £6688 annually when applied to the 12 paramedics now working for the service. These figures have ramifications for the GPOOH service, who cover not only this expense, but the cost of non-prescriber time to gain advice and a potential reduction in patient turnover. 

Future cost–benefit analysis of IP and PGD use should consider the number of times prescriber support is accessed to complete consultations and how this impacts staff timings and patient turnover. However, as previously mentioned, costings should not be the only factor considered, as the additional benefits of employing a prescriber, with the potential to provide more prudent and preventative healthcare, warrants due consideration in any cost–benefit analysis.

### Reasons for gaining prescriber support

Twenty-five per cent of the time, paramedics sought prescriber support when a PGD could have been used independently (n = 22), suggesting clinicians were not as autonomous with PGDs as they could have been. This has been found in another study comparing PGD use to IP (Black et al., 2020). The reasons for this are unclear but are presumably linked to clinician knowledge, confidence and experience.

In some cases, paramedic advice-seeking may indicate enhanced clinical knowledge, by recognising that a non-PGD medication is more appropriate and discussing this with a prescriber. An example of this is highlighted in a survey by Bedson and Latter (2018), where specialist paramedics noted that PGDs were often not in line with local antimicrobial guidance. Clinicians would therefore require prescriber support in order to supply the recommended antibiotic. Electronic patient clinical records do not capture the rationale behind medication selection, so it is impossible to tell how frequently this occurred in the current study.

PGDs can benefit less-experienced staff, however, by providing a safer way to gain experience and confidence in medication provision (Bedson & Latter, 2018). If advice does not diminish with increased knowledge and experience, though, high levels of prescriber support will always be required, suggesting IP is most beneficial for experienced staff.

IP legislation stipulates that prescribing must be within a clinician’s competence level (College of Paramedics, 2018). Therefore, GP advice will be gained when needed. As nurses documented only three occasions where advice was gained (0.7%), the addition of IP appears to increase pharmaceutical knowledge and confidence to almost complete autonomy, supporting findings in previous studies (Black et al., 2020; Courtenay et al., 2015). Bedson and Latter (2018) also found that PGDs may even inhibit clinician autonomy and patient care. It is difficult, however, to ascertain how much of an impact clinician education and experience, availability of GP support and the commonality of patient presentations had on nurse autonomy and support-seeking. A qualitative aspect in future study would provide more valuable data on medication rationale and the impact of clinician confidence and experience.

However, in 62.5% of cases, a PGD could not have been utilised even if the paramedic knew what medication to supply and was confident in doing so, as on most occasions either a PGD medication was excluded from use (28.4%) or there was no PGD for the medication given (34.1%). Consequently, if paramedics in this study had used every opportunity to be autonomous, they would only have been effective 37.5% of the time.

As PGDs are purposefully restrictive to align with legislation (NICE, 2017b), their use will always create a level of dependence on prescribing colleagues. An extensive review by Health Education Northwest (2015) found that prescribers felt their qualification allowed them to complete 95% of consultations independently. With nurse prescribers in this study achieving over this (99.3%), and with the potential for PGD autonomy maxing out at 37.5%, it is clear that paramedics in this setting would have completed more episodes of care independently if they had held a prescribing qualification.

### Limitations

Cross-sectional study designs measure specific variables at one point in time, preventing control or manipulation to limit bias (Bryman, 2016). Unmeasured variables, such as staffing levels, experience, background and education, will have influenced results. Differences are likely to be more prominent in a study comparing two different professions.

Unequal group sizes impacted the results. Variation in the medication provided, frequency of provision and number of advice-seeking episodes may have been underestimated due to the smaller paramedic sample size.

The purposive sample and small number of staff available for inclusion in the study prevented random allocation, limiting generalisability (Cutter, 2012), especially as 130 electronic patient clinical records were excluded from analysis due to missing data.

Due to possible record-keeping inconsistencies, it was unclear if advice-seeking resulted in a written prescription or a verbal order to dispense medication. An accurate record of verbal order and prescription rates would have been useful for future comparison.

Finally, electronic patient clinical records do not capture the rationale behind medication choices. It was impossible to tell if prescriber advice was gained due to not knowing what medication to supply or knowing what to supply but being restricted in doing so, limiting conclusions.

## Conclusion

The study demonstrates that advanced paramedic practitioners working in a GPOOH service can supply a similar level of medication as prescribing nurses by using a combination of PGDs and prescriber support. However, to achieve this, there is substantial reliance on the medical profession.

Nurse prescribers completed almost all consultations autonomously (99.3%). Paramedics, utilising every opportunity to use PGDs autonomously, could only complete up to 37.5% of consultations independently. In view of this, it is clear that advanced paramedic practitioners in this setting would have completed more episodes of care independently if they had held a prescribing qualification, demonstrating a need to prescribe in this service when prescriber support is limited. This is a fundamental consideration for services where verbal order support is not practised, where there are prescriber shortages or where there is a need for a larger, more autonomous workforce. 

For services with good medical availability, PGD use could provide patients with adequate access to medication, while also benefiting less-experienced non-prescribers.

Further research is needed to assess the speed, cost and efficiency of PGD use in comparison with IP.

Finally, it is essential to remember that some benefits of implementing IP cannot be calculated. Managing medicines, providing accessible, preventative and informative healthcare, as well as increasing patient satisfaction and quality of care should be the core focus of any new health initiative and form the building blocks for long-term ill-health reduction and better public health.

## Acknowledgements

We would like to acknowledge the following people: Andrew Oswald, your advice and knowledge allowed us to continue; the Out of Hours Management and IT team for your backing and assistance with data collection; Sue Stevens and Gareth Crabbe for your unwavering support, encouragement, humour and love.

## Author contributions

HS and BM made substantial contributions to conception and design. HS was responsible for acquisition of data. HS and JC were responsible for analysis and interpretation of data. All authors were involved in drafting the manuscript and revising it critically. All authors gave final approval of the version to be published and agreed to be accountable for all aspects of the work in ensuring that questions related to the accuracy or integrity of any part of the work are appropriately investigated and resolved. HS acts as the guarantor for this article.

## Conflict of interest

None declared.

## Ethics

Permission to conduct the study was given by the University Research Committee (reference: 151220). Access to anonymised patient clinical records was given by Health Board Service Management, with all patient, staff and hospital identifiers removed prior to receipt.

## Funding

None.
